# Differences in exercise intensity seems to influence the affective responses in self-selected and imposed exercise: a meta-analysis

**DOI:** 10.3389/fpsyg.2015.01105

**Published:** 2015-08-04

**Authors:** Bruno R. R. Oliveira, Andréa C. Deslandes, Tony M. Santos

**Affiliations:** ^1^Psychiatry and Mental Health Postgraduate Program, Psychiatry Institute, Federal University of Rio de JaneiroRio de Janeiro, Brazil; ^2^Exercise and Sports Sciences Postgraduate Program, Rio de Janeiro State UniversityRio de Janeiro, Brazil; ^3^Physical Education Department, Federal University of PernambucoRecife, Brazil

**Keywords:** pleasure, VO2, autonomy, PRISMA, Feeling Scale, aerobic exercise

## Abstract

Self-selected exercise seems to promote positive affective responses due to the perceived autonomy associated with it. The objective of the present study was to determine the magnitude of differences in Feeling Scale (FS) responses during self-selected and imposed exercise sessions. The PRISMA Statement was adopted for this meta-analysis. The search used PubMed, Scopus, PsycINFO, and ISI Web of Knowledge databases. A total of 10 studies that compared the effects of self-selected and imposed exercise sessions on acute FS responses were included. The screening strategy included: exclusion of studies that were duplicated between databases, abstract screening, and text screening. The standardized mean difference (SMD) between self-selected and imposed exercise sessions categorized in five intensities (equal intensity: both exercises were performed at the same intensity, below lactate/ventilatory threshold (LT/VT): imposed exercise was performed at an intensity below the LT/VT, at LT/VT: imposed exercise was performed at the LT/VT intensity, above LT/VT: imposed exercise was performed at an intensity above the LT/VT, and different intensity: both exercises were performed at different intensities and the intensity of imposed session was not reported relative to LT/VT) and an overall SMD were calculated. Self-selected exercise was used as the reference condition. The subtotal SMD values were as follows: −0.10 (equal intensity), −0.36 (below LT/VT), −0.57 (at LT/VT), −1.30 (above LT/VT), and −0.09 (different intensity) and the overall SMD was −0.41. The results of the present study indicate that the difference between affective responses in self-selected and imposed exercise sessions is dependent on the intensity of the imposed exercise session.

## Introduction

Regular exercise is linked to improvements in several physical and mental factors, and it minimizes the risk of premature mortality and several diseases according to the American College of Sports Medicine (American College of Sports Medicine - ACSM, 2014). Therefore, the development of strategies that promote exercise adherence seems to be important for individual and public health, given the high public health costs (approximately $24 billion) of treating diseases associated with physical inactivity (Schenck et al., 2014).

Several aspects seem to be involved in the decision to maintain an active behavior. In addition to environmental (Rhodes et al., 2009; Coon et al., 2011), socioeconomic and cognitive factors (Rhodes et al., 2009), an exercise prescription is also important for exercise adherence (Dishman and Buckworth, 1996). This assumption is based on the premise that the exercise prescription may influence psychological variables, such as perceived pleasure and enjoyment. It was previously argued that the individual decision to repeat an exercise bout can be influenced by perceived pleasure and enjoyment (Pollock et al., 1978).

Williams et al. (2008) demonstrated that an increase of one unit in the acute Feeling Scale (FS) response induced by exercise is related to an increase of 38 min per week of physical exercise at 6 months of an aerobic exercise program. The FS is an 11-point bipolar scale that is used to monitor affective responses with ranges from −5 (Very bad) to +5 (Very good) including 0 as Neutral (Hardy and Rejeski, 1989). In a review on the subject (Ekkekakis et al., 2011), affective response was indicated as an important motivational determinant of exercise adherence. Furthermore, the American College of Sports Medicine recommended the use of FS as a complementary strategy for monitoring training sessions in its 2011 position stand paper (Garber et al., 2011), which shows the growing scientific interest in monitoring affective responses.

Recently, Ekkekakis (2009b) reported that the self-selection of aerobic exercise intensity may provide superior benefits for affective responses compared to an imposed aerobic exercise intensity. Self-selected exercise sessions are characterized by the individual's freedom to regulate the intensity of the exercise session. However, imposed exercise sessions are characterized by exercise intensity and duration, which are externally defined. The improvement in the affective responses during self-selected exercise may be related to the perceived autonomy of self-selection, which may contribute to positive affective responses (Deci and Ryan, 2000). Additionally, Dishman et al. (1994) mentioned that imposed exercise sessions are usually performed at a constant intensity while self-selected exercise sessions are performed at a freely-chosen intensity. Therefore, both the perceived autonomy (modulated by the exercise mode) and exercise intensity may influence the affective responses. Specifically, the effect of the exercise intensity seems to be linked to the lactate (LT) or ventilatory threshold (VT). Exercise intensities below the LT/VT are related to positive affective responses, while exercise intensities above the LT/VT are related to negative affective responses.

Although it has previously been demonstrated that self-selected exercise may produce benefits in affective responses (Ekkekakis, 2009b), its use may induce an over- or under-estimation of the exercise prescription variables (Johnson and Phipps, 2006). Additionally, previous studies (Parfitt et al., 2006; Rose and Parfitt, 2007; Sheppard and Parfitt, 2008) comparing self-selected and imposed exercise sessions of different intensities have considered imposed sessions with intensities below, at and above the LT/VT. Considering the influence of exercise intensity on affective responses (Reed and Ones, 2006; Reed and Buck, 2009), and that LT and VT may be related to affective responses (Ekkekakis, 2009a), the comparison of the self-selected and imposed exercise sessions at different intensities may lead to misrepresentation of the results. Therefore, the use of different methodological approaches in previous studies may interfere with professional decision-making about the use of self-selected exercise.

The objective of this study was to determine the magnitude of differences between affective responses (measured using FS) of self-selected and imposed exercise sessions according to the different intensities used in the imposed exercise sessions. Considering the effect of intensity on the affective responses, we hypothesize that the effect size would be similar between the FS responses for self-selected and imposed exercise sessions that have equal intensity.

## Materials and methods

The present meta-analysis was performed according to the Preferred Reporting Items for Systematic Reviews and Meta-Analysis, PRISMA Statement (Liberati et al., 2009). The Cochrane recommendations (Higgins and Green, 2011) were used as a complementary guide to perform the present study.

### Protocol and registration

This study was not registered.

### Eligibility criteria

#### Types of studies

Studies, with human participants, that were published in English were included. Theses, conference proceedings and unpublished studies were also included because its inclusion could minimize the risk of bias (Hopewell et al., 2007). No publication date restriction was applied.

#### Participants

Physically active and sedentary participants of both genders and those older than 10 years of age were considered. Active and sedentary participants were included so the results of the present meta-analysis could reflect the results for the general population instead of for a specific population. Studies including participants with any mental or musculoskeletal disorders were excluded.

#### Interventions

The experimental studies (randomized or non-randomized) compared, in a single group of participants, the acute effect of self-selected and imposed exercise sessions on the affective responses that were measured using the FS (Hardy and Rejeski, 1989). To determine the magnitude of differences, the self-selected exercise session was used as the reference condition.

#### Outcomes

The affective response measured using the FS in both exercise conditions (self-selected and imposed) was the outcome of interest for this meta-analysis.

#### Information sources

A database search was performed using PubMed, Scopus, ISI Web of Knowledge and PsycINFO between 16/12/2014 and 17/12/2014. No filters were applied for the search; studies with characteristics differing from the criteria used in this study were excluded after the search was completed. None of the studies from the reference lists of the studies identified in the search were included in this study.

#### Search

The search used the following terms: aerobic exercise AND self-selected AND affective responses, aerobic exercise AND self-selected AND pleasure, physical activity AND self-selected AND affective responses, and physical activity AND self-selected AND pleasure.

#### Studies selection

Studies identified in the above-described search were presented in a spreadsheet (Microsoft Corporation, Redmond, USA) with columns that are labeled *title, abstract*, and *database*. The following screening criteria were applied: a. screening for duplicates (studies found in more than one database were excluded so that we only evaluated one record of each study); b. screening of abstracts (studies that did not compare the exercise conditions or outcomes previously specified in the present meta-analysis were excluded); c. screening of the text (studies that did not meet the aforementioned criteria or did not report measures of central tendency and/or variability of the FS measurements for both self-selected and imposed exercise were excluded).

### Data selection

Characteristics of participants (n, age, sex, and VO_2Max_), exercise conditions (intensity, duration, and ergometer), and mean and standard deviation values for the FS in each study were recorded. For the studies using subgroups in the comparison matrix (e.g., sedentary and active), the mean FS values of the pre, in and post-task were calculated for each condition (self-selected and imposed), regardless of groups. Data were extracted from the text, tables and/or figures of the selected studies. When the results were reported in figures, the data were retrieved using the “horizontal and vertical dimension” tool of Corel Draw software (CorelDRAW, GraphicsSuite, version 16.0 for Windows). Values were extracted in millimeters and then converted to real values on the FS using the cross-multiplication method.

### Risk of bias

The authors of the present study developed a scale to quantify the methodological quality of the selected studies. This strategy has been suggested by The Cochrane Collaboration Group (Higgins and Green, 2011) and was adopted because the selected studies had specific characteristics not reported on scales readily available in the literature. The risk of bias in individual studies was assessed through a visual analysis of a funnel plot graph, and the risk of bias across studies was assessed using heterogeneity results.

### Summary measures

The analyses were performed considering different intensity categories of the imposed condition. The categories were as follows: equal intensity (imposed and self-selected sessions performed at equal intensities); below LT/VT intensity (imposed and self-selected sessions performed at different intensities and the imposed session was performed at an intensity below the LT/VT); at LT/VT intensity (imposed and self-selected sessions performed at different intensities and the imposed session performed at the same intensity as LT/VT); above LT/VT intensity (imposed and self-selected sessions performed at different intensities and the imposed session performed at an intensity above the LT/VT), and different intensity (when imposed and self-selected sessions were performed at different intensities, although the imposed session intensity was not reported relative to LT/VT). The LT/VT reported in the selected studies refers to the second threshold and was used as the marker for defining the intensity categories. Although the LT and VT did not always occur at the same intensity, both may be used as markers for affective responses. Therefore, the LT and VT were interchangeably reported as the markers for the exercise intensity in the present meta-analysis. Studies using imposed sessions of different intensities (e.g., below and above the LT/VT) were reported in each of the categories that were compared. A random-effect was used since that heterogeneity is expected due to the differences in methodological approach of studies. The standardized mean difference (SMD) was calculated using Equation 1 and considered the mean value of the in-task and post-task FS responses for each study. The heterogeneity test and funnel plot were performed to detect bias. The analyses were performed using Stata software v. 11.0 (StataCorp LP, College Station, USA). The SMD was interpreted as suggested by Cohen (1988) using the following classification: 0.20 to 0.49 = Small; 0.50 to 0.79 = Moderate; or > 0.79 = Large. Values lower than 0.20 were called as Trivial since that no classification was attributed by Cohen (1988) to these values.

(1) $$\begin{array}{l} \text{SMD}=\frac{\left(M_{imp}-M_{ss}\right)}{\sqrt{\frac{\left({{SD}_{ss}}^{2}+{{SD}_{imp}}^{2}\right)}{2}}} \end{array}$$

Where: *M*_*ss*_ = mean of self-selected; *M*_*imp*_ = mean of imposed; *SD*_*ss*_ = standard deviation of self-selected; and *SD*_*imp*_ = standard deviation of imposed.

## Results

### Study selection and characteristics

**Figure 1:**
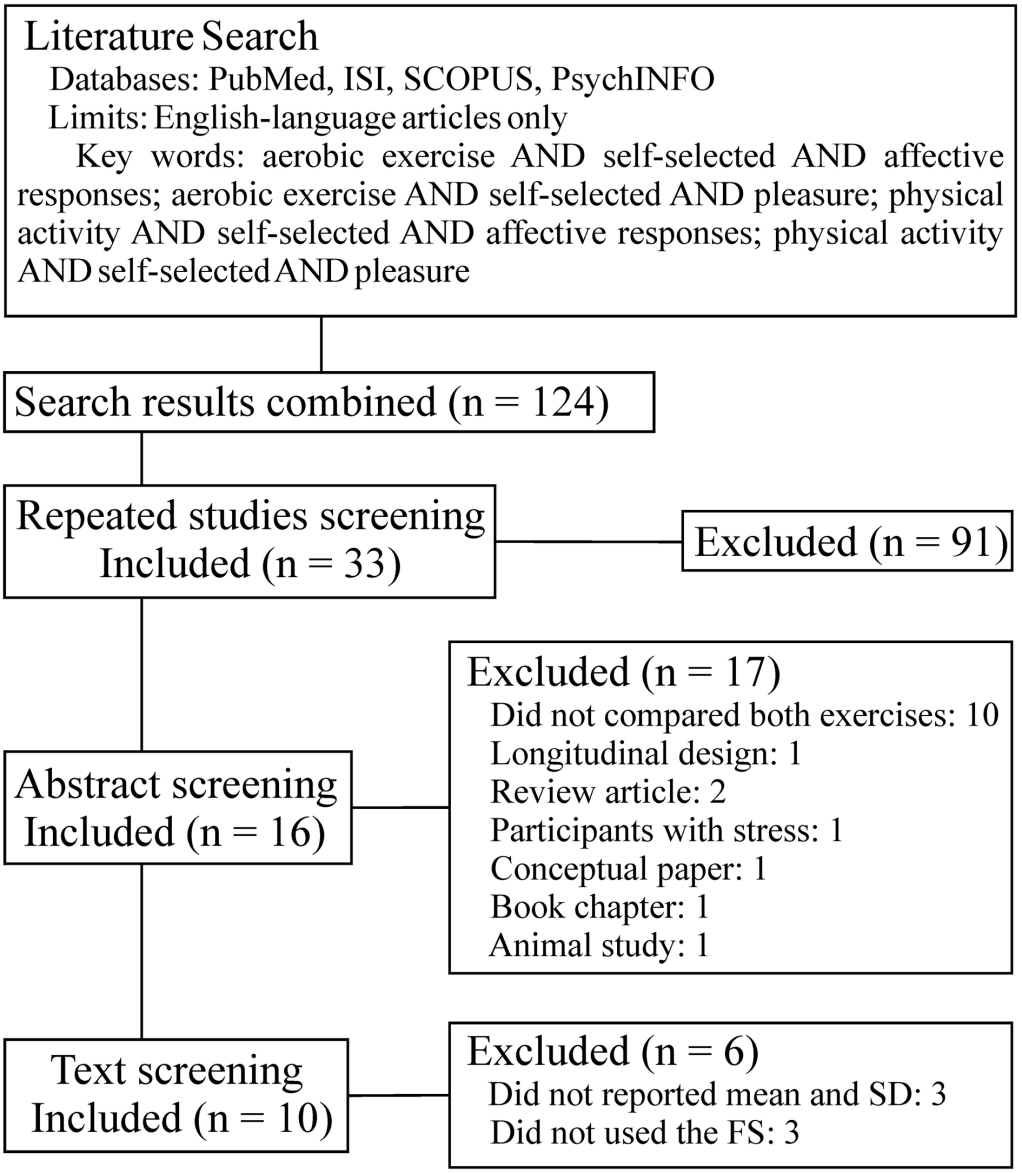
**Flow diagram of study selection**.

A total of 124 studies were retrieved from all databases. After the application of the exclusion criteria, a total of 10 studies were considered as eligible and reviewed in a meta-analysis fashion (Ekkekakis and Lind, 2006; Parfitt et al., 2006; Rose and Parfitt, 2007, 2012; Sheppard and Parfitt, 2008; Stych and Parfitt, 2011; Haile et al., 2013; Williams and Raynor, 2013; Hamlyn-Williams et al., 2014; Oliveira et al., 2015). The description of the screening steps is presented in Figure 1.

### Summary of studies

The selected studies had a total of 241 participants. The mean age ranged from 12.5 ± 0.5 (Stych and Parfitt, 2011) to 45.1 ± 10.1 years (Rose and Parfitt, 2012) and the VO_2Peak_ ranged from 23.3 ± 5.3 (Ekkekakis and Lind, 2006) to 48.7 ± 9.7 mL.kg^−1^.min^−1^ (Oliveira et al., 2015). With respect to the exercise volume, two studies (Sheppard and Parfitt, 2008; Stych and Parfitt, 2011) reported a 15-min duration, five studies (Ekkekakis and Lind, 2006; Parfitt et al., 2006; Rose and Parfitt, 2007; Haile et al., 2013; Hamlyn-Williams et al., 2014) reported a 20-min duration, one study (Rose and Parfitt, 2012) reported a 30-min duration, one study (Williams and Raynor, 2013) reported a 1/3 mile distance and one study reported a mean duration of 38.9 min. Six studies (Ekkekakis and Lind, 2006; Parfitt et al., 2006; Rose and Parfitt, 2007, 2012; Williams and Raynor, 2013; Hamlyn-Williams et al., 2014) were conducted on a treadmill while four studies (Sheppard and Parfitt, 2008; Stych and Parfitt, 2011; Haile et al., 2013; Oliveira et al., 2015) were conducted on a cycle ergometer. The characteristics of the studies are presented in Table 1. A qualitative analysis of the selected studies was performed. Five studies (Rose and Parfitt, 2012; Haile et al., 2013; Williams and Raynor, 2013; Hamlyn-Williams et al., 2014; Oliveira et al., 2015) compared the self-selected and imposed exercise with the same intensity. In only two studies (Hamlyn-Williams et al., 2014; Oliveira et al., 2015), the pace was continuously adjusted during the self-selected exercise, which may increase the external validity considering that participants may continuously adjust their pace in a real-world scenario. The methodological quality scale of the selected studies containing all quality criteria is presented in Table 2.

**Table 1:** **Characteristics of selected studies**.

**Study**	**Participants**			**Conditions**					**Volume**	**Ergometer**
	***N***	**Age**	**VO_2Peak_ (mL.kg^−1^.min^−1^)**	**Intensity variable**	**Self-selected intensity**	**Imposed intensity**				
Parfitt et al., 2006	12 men	36.5 (10.5)	34.1 (5.1)	HR (bpm)	120.7 (18.5)	Below LT		104.4 (10.9)	20 min	Treadmill
						Above LT		135.9 (13.6)		
Ekkekakis and Lind, 2006^*^	25 women	43.3 (4.8)	23.3 (5.3)	Speed (m.s^−1^)	1.67 (0.43)	1.85 (.45)			20 min	Treadmill
Rose and Parfitt, 2007	19 women	39.4 (10.3)	36.1 (3.0)	%HR_*Max*_	68.1 (6.7)	Below LT		69.1 (3.0)	20 min	Treadmill
						At LT		80.6 (3.5)		
						Above LT		89.3 (3.3)		
Sheppard and Parfitt, 2008	22 adolescents	13.3 (0.3)	43.3 (6.0)	Watts	83 (22)	Below LT		75 (15)	15 min	Cycle ergometer
						Above LT		121 (21)		
Stych and Parfitt, 2011	26 adolescents	12.5 (0.5)	36.1 (6.9)	Watts	57.6 (13.9)	Below VT		55 (8)	15 min	Cycle ergometer
						At VT		NR		
						Above VT		NR		
Rose and Parfitt, 2012^*^	32 women	45.1 (10.1)	35.7 (4.8)	%HR_Max_	74.8 (8.0)	75.4 (10.2)			30 min	Treadmill
Haile et al., 2013^*^	32 men	22.3 (2.2)	41.7 (7.4)	%VO_2peak_	57.0 (10.8)	57.0 (10.8)			20 min	Cycle ergometer
Williams and Raynor, 2013	29 women	39.7 (12.3)	NR	%HR_Max_	55.8 (7.6)	Equal to Self-selected		54.8 (6.6)	1/3 mile	Treadmill
						20% > Self-selected		58.3 (7.9)		
Hamlyn-Williams et al., 2014	27 adolescents	14.6 (0.8)	42.9 (7.7)	%VO_2_-at-VT	93.0 (11.7)	90.9 (11.0)			20 min	Treadmill
Oliveira et al., 2015	17 men	31 (7)	48.7 (9.7)	Watts	150.8 (49.0)	149.6 (48.4)			38.9 (14.8)	Cycle ergometer

* Studies that compared different groups, for these studies average values of groups in self-selected and imposed conditions were extracted; NR, not reported; HR, heart rate; VO_2Peak_, peak oxygen consumption; LT, lactate threshold; and VT, ventilatory threshold.

**Table 2:** **Qualitative characteristics of selected studies**.

**Study**	**FS measured pre, during and post exercise session**	**Self-selected and imposed sessions applied in a randomized order**	**Equalization of self-selected and imposed exercise sessions**	**Continuous free pace adjustment during self-selected session**	**Meet the sample size estimation or the sample size is ≥ than the average sample size of all studies (*n*≥24)**	**Total**
Parfitt et al., 2006	1	1	0	0	0	2
Ekkekakis and Lind, 2006	0	0	0	0	1	1
Rose and Parfitt, 2007	1	1	0	0	0	2
Sheppard and Parfitt, 2008	1	0	0	0	0	1
Stych and Parfitt, 2011	1	0	0	0	0	1
Rose and Parfitt, 2012	1	1	1	0	1	4
Haile et al., 2013	1	0	1	0	1	3
Williams and Raynor, 2013	1	1	1	0	1	4
Hamlyn-Williams et al., 2014	0	1	1	1	1	4
Oliveira et al., 2015	1	0	1	1	0	3

### Standardized mean difference

**Figure 2:**
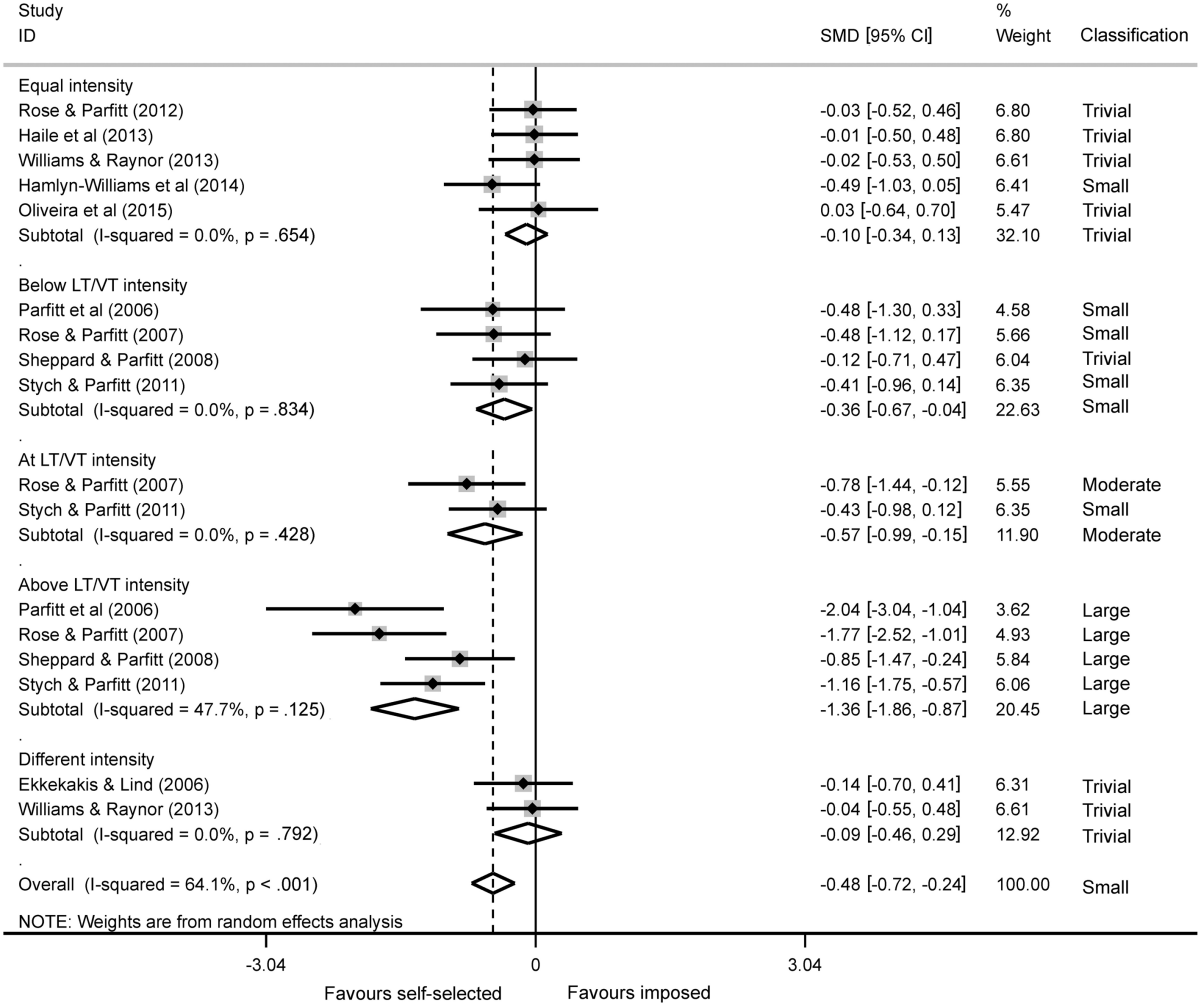
**Forest Plot containing the subtotal effects for each intensity category and the overall effect for all intensities**. The vertical solid line represents the self-selected exercise as the reference condition, and the vertical dashed line represents the overall effect observed in the present meta-analysis.

The present meta-analysis considered different intensity categories for the comparison. Five studies (Rose and Parfitt, 2012; Haile et al., 2013; Williams and Raynor, 2013; Hamlyn-Williams et al., 2014; Oliveira et al., 2015) were included in the “equal intensity” category, four studies (Parfitt et al., 2006; Rose and Parfitt, 2007; Sheppard and Parfitt, 2008; Stych and Parfitt, 2011) were included in the “below LT/VT” intensity category, two studies (Rose and Parfitt, 2007; Stych and Parfitt, 2011) were included in the “at LT/VT” intensity category, four studies (Parfitt et al., 2006; Rose and Parfitt, 2007; Sheppard and Parfitt, 2008; Stych and Parfitt, 2011) were included in the “above LT/VT” intensity category, and two studies were included in the “different intensity” category (Ekkekakis and Lind, 2006; Williams and Raynor, 2013). Both the subtotal and overall SMD were provided for each intensity category and for all intensity categories combined respectively. The mean and standard deviation values of FS used to calculate the SMD are presented in Table 3. The subtotal SMD classifications ranged from Trivial to Large, and the overall SMD was classified as Small (Figure 2), indicating that self-selected exercise provided higher FS responses than the imposed exercise. However, when subtotal SMD values are considered it is possible to verify that the imposed exercise intensity influences the FS responses. For example, when the exercise intensity of the self-selected and imposed exercise was equal, the subtotal SMD was Trivial, which supports the hypothesis of the present study.

**Table 3:** **Mean and SD values of Feeling Scale of the studies used in meta-analysis**.

**Study**	**Condition**				
	**Self-selected**		**Imposed**		
	**Mean**	***SD***	**Intensity category**	**Mean**	***SD***
Parfitt et al., 2006	3.75	0.76	Below LT/VT intensity	3.22	1.35
			Above LT/VT intensity	0.83	1.88
Ekkekakis and Lind, 2006	2.42	0.28	Different intensity	2.38	0.29
Rose and Parfitt, 2007	2.83	0.98	Below LT/VT intensity	2.33	1.12
			At LT/VT intensity	1.90	1.38
			Above LT/VT intensity	0.52	1.57
Sheppard and Parfitt, 2008	3.27	1.80	Below LT/VT intensity	3.05	1.79
			Above LT/VT intensity	1.71	1.85
Stych and Parfitt, 2011	3.89	1.21	Below LT/VT intensity	3.32	1.54
			At LT/VT intensity	3.31	1.48
			Above LT/VT intensity	2.21	1.65
Rose and Parfitt, 2012	3.05	1.34	Equal intensity	3.02	1.25
Haile et al., 2013	2.11	1.44	Equal intensity	2.09	1.43
Williams and Raynor, 2013	2.67	1.75	Equal intensity	2.64	1.68
			Different intensity	2.60	1.90
Hamlyn-Williams et al., 2014	1.89	1.71	Equal intensity	0.97	2.02
Oliveira et al., 2015	2.04	1.79	Equal intensity	2.10	1.94

### Risk of bias

**Figure 3:**
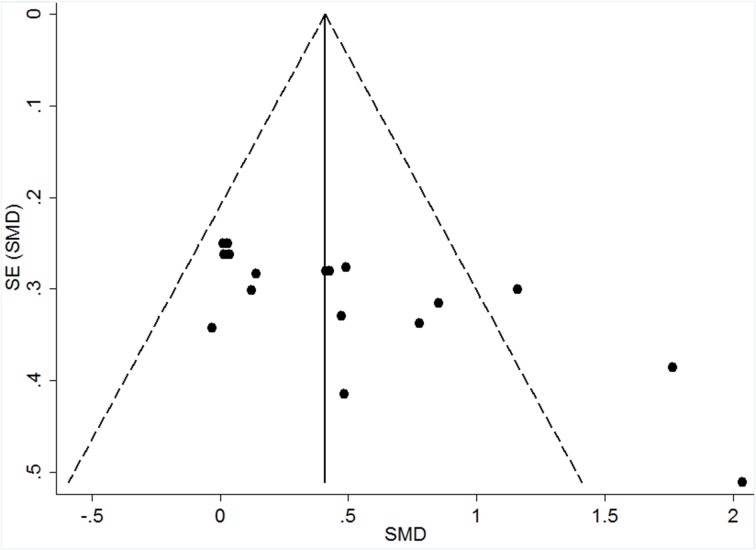
**Funnel plot considering all studies (overall asymmetry)**. The dashed line represents the pseudo CI_95%_. SE, standard error; and SMD, standardized mean difference.

The visual analysis of the funnel plot showed data asymmetry, with three studies (Parfitt et al., 2006; Rose and Parfitt, 2007; Stych and Parfitt, 2011) in the above LT/VT intensity category falling outside the pseudo-confidence interval (CI_95%_), as reported in Figure 3. Heterogeneity was only observed for the overall SMD, with *I*^2^ = 64.1% and *p* < 0.001 (Figure 2). These results are likely due to the differences in the exercise intensities applied in the imposed exercise condition, which could have contributed to a higher variance in the FS responses between studies.

## Discussion

### Summary

The present meta-analysis reviewed the affective responses of self-selected (in which the exercise intensity is regulated by the individual) and imposed exercise sessions (in which the exercise intensity is regulated externally, usually by a fitness professional) based on different intensities of the imposed sessions. The overall effect of the exercise mode was Small and indicated better affective responses in the self-selected exercise. However, the subtotal effects observed for each intensity category have also to be considered. These subtotal effects showed that the exercise intensity played a key role in the affective responses. When self-selected and imposed exercise sessions were performed at equal intensities, the effect size for affective responses was Trivial, while for the other intensity categories (in which the exercise intensity of self-selected and imposed exercise sessions were different) the affective responses were different, especially in the “above LT/VT” intensity. In a review, Ekkekakis discussed the benefits of self-selected exercise on affective responses presenting its mechanisms (Ekkekakis, 2009b). Differently, the present study suggests that the exercise mode (self-selected or imposed) is not the main factor for affective responses considering that a Trivial effect was observed when the self-selected and imposed exercise intensities were equal. Therefore, exercise intensity seems to have a greater impact on the affective responses than the exercise mode. It is important to consider that all studies comparing self-selected and imposed exercise with the same intensity were published after Ekkekakis' review. In another review study (Ekkekakis et al., 2011), the authors reported an inverse relationship between exercise intensity and affective responses. Considering this premise, the results in the present meta-analysis are in agreement considering that imposed exercise performed at intensities above the LT/VT (above LT/VT intensity category) had more negative affective responses than self-selected exercise which was performed at lower intensities.

### Explanations

Although the mechanisms are not well established (Ekkekakis et al., 2011), the Dual-Mode Theory seems to better explain the observed pattern of affective responses. According to this theory, there is a predominance of cognitive factors (associated with pleasurable sensations) in exercise sessions with intensities below the LT/VT, while there is a predominance of interoceptive factors (associated with unpleasant sensations) in exercise sessions with intensities above the LT/VT (Ekkekakis, 2009a). Future studies should investigate the relationship between exercise intensity and affective responses in order to establish the pattern of affective responses to a larger range of intensities than the traditionally investigated based on LT/VT.

Some studies have not directly tested the hypothesis that self-selected exercise is better than imposed exercise in promoting positive affective responses. For example, three of the selected studies (Parfitt et al., 2006; Rose and Parfitt, 2007; Stych and Parfitt, 2011) tested the Dual-Mode Theory. Although, independent of the objectives of previous studies, the available literature on this subject may influence decision-making for the use of self-selection. Thus, this meta-analysis has practical implications demonstrating that exercise intensity may influence affective responses more than the exercise mode (self-selected or imposed exercise). Although it has been previously demonstrated that the acute affective response (measured using the FS) can predict participation in aerobic training programs (Williams et al., 2008), we must note that all original studies used in this meta-analysis measured only acute affective responses. Therefore, the chronic effect of self-selected and imposed exercise sessions on affective responses remains unclear. It is possible to hypothesize that an imposed exercise program in which the exercise intensity is higher than the self-selected level would result in greater physiological adaptations. The attainment of greater physiological adaptations could induce increased self-efficacy or motivation. Both aspects (self-efficacy and motivation) seem to be important in exercise adherence (Deci and Ryan, 2000). Therefore, acute and chronic psychological responses may influence exercise adherence.

While the present results suggest that both self-selected and imposed exercise sessions can induce positive affective responses, it is necessary to consider that self-selection may be linked to an intensity in which the participants achieve a personal “optimal physiological adjustment”, resulting in an “optimal affective state”. Therefore, fitness professionals could use self-selection to determine this optimal physiological adjustment and achieve the optimal affective state for each individual. The results presented in this meta-analysis do not explain the mechanisms underlying the relationship between the self-selection of exercise and the “optimal affective state.” An individual who undergoes a self-selected exercise session might be able to determine an exercise intensity that is compatible with his/her personal optimal affective state. However, this hypothesis should be tested in future studies.

### Limitations

Some factors should be considered in the interpretation of the present study. Although this possibility is described by the *PRISMA Statement* (Liberati et al., 2009), the scale used for determining the quality of the studies is based on the arbitrary criteria of these authors and carries a possibility of bias. This limitation may be attributed to the difficulty in assigning the criteria that should be included in the scale. The Cochrane group (Higgins and Green, 2011) discourages the use of quality scales for study selection; however, considering that our scale was not used in the study selection, the limitations on the use of a scale were minimized. However, the authors emphasize that the scales available in the literature do not include criteria that are relevant for the conditions investigated in this meta-analysis. The studies included in the meta-analysis presented with heterogeneity and funnel plot asymmetry, which could indicate bias; however, this seems to be the result of the different exercise intensities used in the studies, which in turn could lead to high variability of affective responses. Some terms used in the study search are not included in the MeSH database, which may make it difficult to repeat the study. Some studies that compared self-selected exercise to two or more different imposed exercise conditions (Parfitt et al., 2006; Rose and Parfitt, 2007; Sheppard and Parfitt, 2008; Stych and Parfitt, 2011; Williams and Raynor, 2013) were included in different subgroups of the meta-analysis which may result in the inflation of the precision of effect estimates (Higgins and Green, 2011).

## Conclusions

In summary, the results of this meta-analysis indicate that exercise intensity is the greatest determinant of affective responses compared to exercise mode (self-selected and imposed). It is reasonable to assume that if exercise mode is the main factor influencing affective responses (comparing self-selected to imposed exercise) independent of exercise intensity, the “equal intensity” condition would demonstrate better affective responses in the self-selected exercise condition, based on the premise of higher perceived autonomy during self-selected exercise. The results of this study reinforce the premise of the Dual-Mode Theory; however, chronic studies should be conducted to investigate the effect of self-selected and imposed exercise on adherence to exercise program over time.

### Conflict of interest statement

The authors declare that the research was conducted in the absence of any commercial or financial relationships that could be construed as a potential conflict of interest.
